# Angiogenesis and radiological tumor growth in patients with glioblastoma

**DOI:** 10.1186/s12885-018-4768-9

**Published:** 2018-09-03

**Authors:** Vilde Elisabeth Mikkelsen, Anne Line Stensjøen, Unn Sophie Granli, Erik Magnus Berntsen, Øyvind Salvesen, Ole Solheim, Sverre Helge Torp

**Affiliations:** 1 Department of Clinical and Molecular Medicine, Faculty of Medicine and Health Sciences, NTNU - Norwegian University of Science and Technology, Erling Skjalgssons gate 1, 7030 Trondheim, Norway; 2 Department of Circulation and Medical Imaging, Faculty of Medicine and Health Sciences, NTNU - Norwegian University of Science and Technology, Trondheim, Norway; 3 Department of Neurosurgery, St. Olavs University Hospital, Trondheim, Norway; 4 Cellular and Molecular Imaging Core Facility (CMIC), Faculty of Medicine and Health Sciences, Norwegian University of Science and Technology (NTNU), Trondheim, Norway; 5 Department of Radiology and Nuclear Medicine, St. Olavs University Hospital, Trondheim, Norway; 6 Department of Public Health and Nursing, Faculty of Medicine and Health Sciences, NTNU - Norwegian University of Science and Technology, Trondheim, Norway; 7 National Advisory Unit for Ultrasound and Image Guided Therapy, St. Olavs University Hospital, Trondheim, Norway; 8 Department of Neuromedicine and Movement Science, Faculty of Medicine and Health Sciences, NTNU - Norwegian University of Science and Technology, Trondheim, Norway; 9 Department of Pathology, St. Olavs University Hospital, Trondheim, Norway

**Keywords:** Angiogenesis, Glioblastoma, Histopathology, Magnetic resonance imaging, Microvessel density, Tumor growth, Tumor biology, Tumor hypoxia

## Abstract

**Background:**

The preoperative growth of human glioblastomas (GBMs) has been shown to vary among patients. In animal studies, angiogenesis has been linked to hypoxia and faster growth of GBM, however, its relation to the growth of human GBMs is sparsely studied. We have therefore aimed to look for associations between radiological speed of growth and microvessel density (MVD) counts of the endothelial markers vWF (Factor VIII related antigen) and CD105 (endoglin).

**Methods:**

Preoperative growth was estimated from segmented tumor volumes of two preoperative T1-weighted postcontrast magnetic resonance imaging scans taken ≥14 days apart in patients with newly diagnosed GBMs. A Gompertzian growth curve was computed from the volume data and separated the patients into two groups of either faster or slower tumor growth than expected. MVD counts of the immunohistochemical markers von Willebrand factor (vWF) (a pan-endothelial marker) and CD105 (a marker of proliferating endothelial cells) were assessed for associations with fast-growing tumors using Mann-Whitney U tests and a multivariable binary logistic regression analysis.

**Results:**

We found that only CD105-MVD was significantly associated with faster growth in a univariable analysis (*p* = 0.049). However, CD105-MVD was no longer significant when corrected for the presence of thromboses and high cellular density in a multivariable model, where the latter features were significant independent predictors of faster growth with respective odds ratios 4.2 (95% confidence interval, 1.2, 14.3), *p* = 0.021 and 2.6 (95% confidence interval, 1.0, 6.5), *p* = 0.048.

**Conclusions:**

MVDs of neither endothelial marker were independently associated with faster growth, suggesting angiogenesis-independent processes contribute to faster glioblastoma growth.

**Electronic supplementary material:**

The online version of this article (10.1186/s12885-018-4768-9) contains supplementary material, which is available to authorized users.

## Background

Glioblastoma (GBM) is the most common primary malignant brain tumor in adults [1], with a median overall survival of only 10 months in unselected patients [2]. GBMs are characterized by a highly heterogenous histopathology [3–5], a high secretion of pro-angiogenic factors [6, 7], extensive vascularity [8, 9], and rapid pretreatment growth [10, 11]. The pretreatment growth has been shown to vary considerably among patients [10] and slower growth to be an independent predictor of long term survival in patients with GBM [12].

It is of major interest to understand biological processes behind the variations in speed of growth observed in human GBMs, which in turn could reveal future targets of therapies hampering growth. We have recently studied relations between histopathological features and radiological speed of pretreatment tumor growth [13]. We found that thromboses and high cellular density were significant independent predictors of faster preoperative tumor growth [13]. These findings are in line with hypotheses suggesting thrombosis as an initiator of hypoxia facilitating outward tumor expansion, plausibly through stimulation of angiogenesis [9, 14]. Neovascularization is fundamental for the survival and expansion of tumors [15], and angiogenesis has been extensively linked to hypoxia and growth of GBM in animal studies [16–19]. However, in randomized trials, antiangiogenic therapy has not shown any significant survival benefit in GBMs [20, 21]. Still, the degree of angiogenesis has not been assessed for relations with radiological speed of pretreatment growth in human GBMs.

In a cohort of 102 GBMs previously assessed for radiological speed of growth [10], we sought to investigate possible associations between pretreatment speed of tumor growth and the degree of angiogenesis quantified by microvessel density (MVD) measurements. The MVDs were immunohistochemically assessed by means of two endothelial markers: von Willebrand factor (vWF or FVIII related antigen), a pan-endothelial marker [22], which illustrates the metabolic demand of the tumor [23]; and endoglin (CD105), a marker of proliferating endothelial cells [24], which reflects the degree of angiogenesis [23]. In addition, we investigated the correlation between the MVDs and their associations with the histopathological features thromboses, high cellular density, high vascular density, and mitotic count.

## Methods

### Inclusion and exclusion criteria

As previously described, patients were retrospectively selected from all newly diagnosed GBM patients ≥18 years of age operated at St Olavs Hospital – Trondheim University Hospital, Norway between January 2004 and May 2014 (262 patients) [10]. Selection criteria were ≥ 2 pretreatment T_1_-weighted postcontrast magnetic resonance imaging (MRI) scans separated by ≥14 days and histopathologically verified GBMs according to the 2007 World Health Organization (WHO) Classification [25]. Exclusion criteria were non-contrast-enhancing tumors and gliomatosis cerebri (defined by radiological criteria [26]). In addition, four cases were excluded because of insufficient tissue amount or morphology for the MVD assessments, which left 102 patients eligible for further analyses.

### Volume segmentation and growth rates

The segmentation of tumor volumes and establishment of growth rates have previously been described in detail [10]. The volume segmentation was performed by ALS and controlled by EMB (a neuroradiologist) using the software BrainVoyager QX (Brain Innovation, Maastricht, The Netherlands). Both preoperative MRI scans were segmented for total tumor volumes, defined as the combined volume of the non-contrast-enhancing central part (i. e. necrosis) and the contrast-enhancing rim. In addition, the reproducibility of the tumor volume assessments has been assessed and concluded as satisfactory [10].

**Fig. 1:**
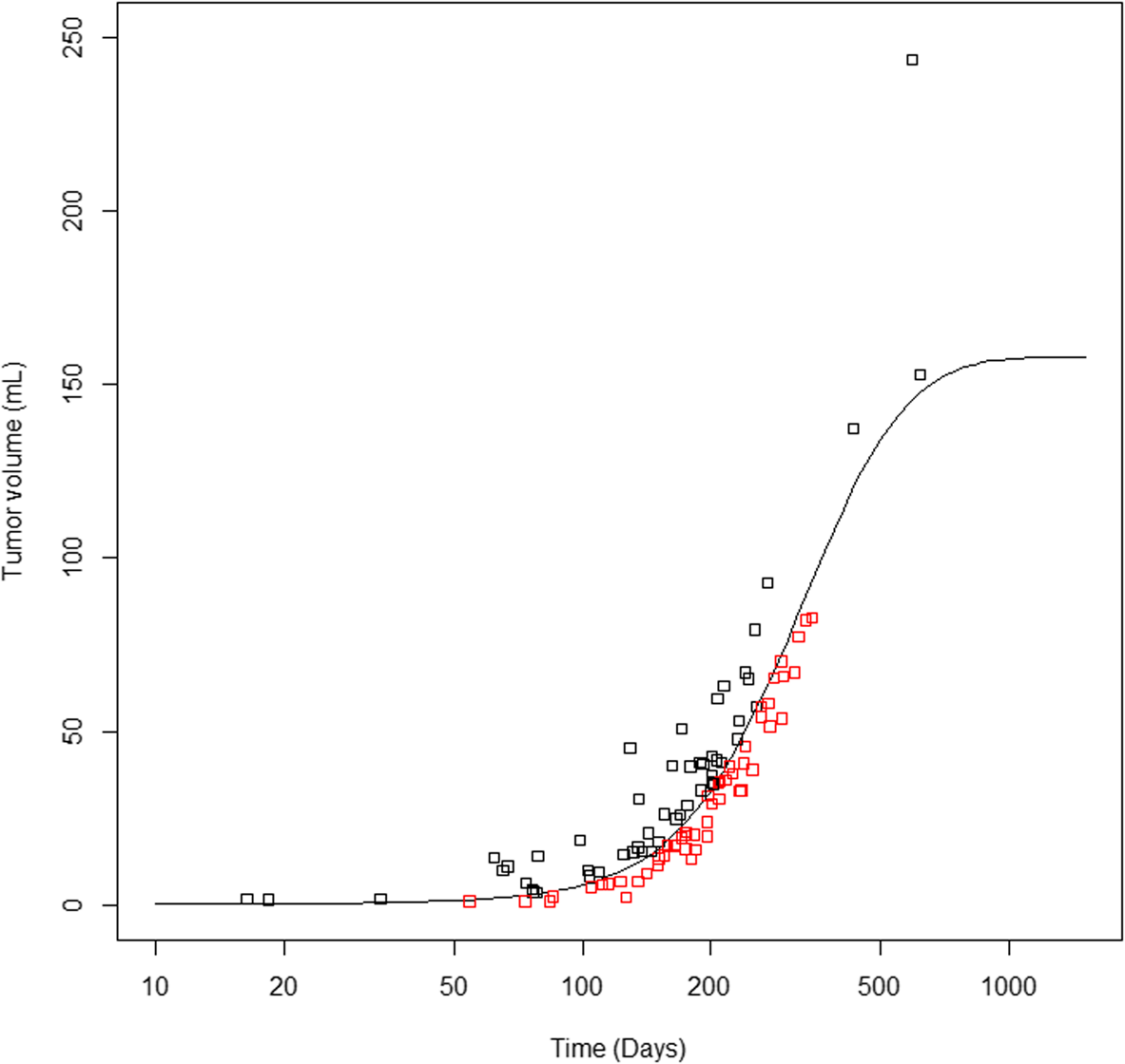
Gompertzian growth pattern. The expected Gompertzian growth curve computed from segmented tumor volumes of two preoperative MRI scans and the interval between them in 106 patients [10]. Time is presented as a logarithmic scale. The squares represent tumor volumes at the second MRI scans: the black squares are tumor volumes with a larger increase in size than expected from their initial volume (fast-growing tumors), while the red squares are tumors with a smaller volume increase than expected from the curve (slow-growing tumors). For illustration purposes, the curve was drawn from a tumor with an arbitrary size of 0.135 mL at day 0

The fitness of different growth patterns based on the segmented tumor volumes and the time intervals between the scans, have previously been assessed [10]. The Gompertzian growth pattern (Fig. 1) was concluded as the most biologically reasonable growth pattern [10], and all tumors were assumed to follow this growth pattern. Since growth rates were highly dependent upon tumor volume [10], a point estimate (such as doubling time) would be a wrong representation of tumor growth. To account for this issue, we calculated an expected Gompertzian growth curve based on the volume data from 106 patients [10]. The curve dichotomized the patients into having tumors with a larger or smaller volume increase than expected from the curve (i.e. fast-growing or slow-growing tumors) (Fig. 1) [10]. These two groups have previously been shown to associate with survival of GBM patients [12]. In the current study, these groups were assessed for associations with the MVDs.

### Histopathology

All routine hematoxylin-eosin (HE) sections for each case have previously been microscopically assessed by VEM (a medical research student) and controlled by SHT (an experienced neuropathologist) for the presence of 30 different histopathological features, which have previously been described in detail [13]. These features were assessed for associations with the same groups of growth, and thromboses, high cellular density, vascular density, and mitotic count were of interest to this study. Thromboses were defined as vascular structures partly or completely occluded with fibrin. The general cellular and vascular densities of viable tumor areas were subjectively graded as low, moderate, or high. For statistical analyses, the 2 lowest categories were merged, because very few cases were graded as low in both variables. Mitoses were counted in 10 high-power fields (HPFs) in areas of highest mitotic counts (hotspots).

### Immunohistochemistry

From each patient, one representative tumor sample from formalin-fixed paraffin-embedded (FFPE) tissue were cut at 4 μm, dried, deparaffinized, and rehydrated. Of the 102 cases, 11 cases had sections from FFPE frozen tissue. We applied vWF (vWF, 1:2000, polyclonal rabbit, EnV+/HRP, Dako Denmark AS, Glostrup, Denmark) and CD105 (CD105, SN6h, 1:50, monoclonal mouse, LSAB/HRP, Dako Denmark AS, Glostrup, Denmark). Optimum antibody concentrations were determined by titrations. For CD105, antigen retrieval was achieved with proteolytic enzymes, endogenous peroxidase activity was quenched with peroxidase block, and sections were incubated with the primary antibody for 24 h at 4 °C with cover glass after pretreatment with serumfree proteinblock. Both endothelial markers had negative controls and positive internal controls.

### Quantification of microvessel densities

MVDs of vWF and CD105 were assessed by VEM, who was blinded to the growth data. A Nikon Eclipse 80i microscope and a Nikon CFI 10×/22 grid at × 400 magnification (area within the grid equal to 0.059 mm^2^) were used for the MVD assessments. MVDs were assessed after the methods of Weider et al. [27] with some modifications. MVDs were computed as the mean count of vessels within the grid for three HPFs of highest vascular densities (i. e. hotspots). Hotspots were identified by scanning using × 4 and × 10 objectives on vWF sections; corresponding hotspots were identified on CD105 sections. Only areas with ≥50% of viable central tumor tissue were counted. Tissue edges and areas with excessive hemorrhage and/or desmoplasia were avoided. Any individually stained endothelial cell or vessel within or in contact with the grid were counted. Moreover, because of the heterogeneous morphology of GBM vessels [28], each lumen was counted for long branched vessels and glomeruloid tufts as described by Kraby et al. [29]. In addition, separate units of ≥2 staining endothelial cells within the same vascular structure were counted as one vascular unit. Altogether, one case was not assessed for vWF-MVD due to high background staining, and another case was not evaluated for CD105-MVD due to non-existent antigenicity.

### Statistical analyses

The estimation of growth rates and curves were computed using R version 2.13.1 [10] and analyses involving histopathology and MVDs were performed using IBM SPSS Statistics 24. Statistical significance was defined as *p* < 0.05 without corrections for multiple testing. The correlation between vWF-MVD and CD105-MVD was assessed using the Spearman rank correlation test. Associations between both MVDs and histopathological features were assessed using Mann-Whitney U tests (categorical vs continuous) and Spearman rank correlation tests (continuous vs continuous). vWF-MVD and CD105-MVD were further investigated for associations with fast-growing tumors using Mann-Whitney U tests. Finally, CD105-MVD was included in a multivariable binary logistic regression model with the histopathological features previously found to be significantly associated with faster growth in the same patients (thromboses, high cellular density, and mitotic count) [13]. Mitotic count was excluded from the model due to significant associations with all other features included [13].

## Results

### Patient characteristics

Of the 102 included patients, 69% (70 patients) were male. The mean age was 63 years, range 26–83 years old. All cases were immunopositive for glial fibrillary acidic protein (GFAP) and two for isocitrate dehydrogenase mutation (IDH1-R132H) [12]. Eighty-two patients (80%) were preoperatively treated with corticosteroids. The median tumor volume was 17.7 mL (range 0.05–146.45 mL) from the first MRI scans and 27.5 mL (range 0.98–243.52 mL) from the second MRI scans. The median interval between the scans was 22.5 days, range 14–98 days. Overall, 52 patients (51%) had fast-growing tumors, and patient characteristics within the growth groups have previously been reported [12].

### Descriptive data

**Fig. 2:**
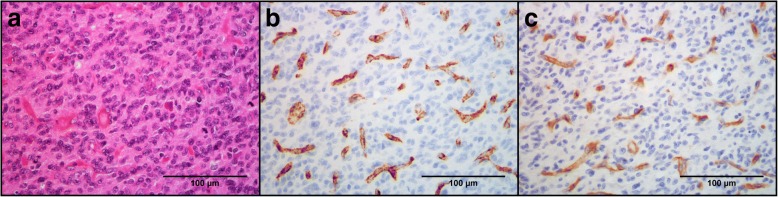
Vascular structures at HE, vWF, and CD105 stains. Pictures are taken from corresponding HPFs in the same tumor of an area of high vascular density at × 400 magnification. **a** HE stain. Plenty of visible small vascular structures in an area of central tumor with small cell morphology. **b** vWF stain. Granular cytoplasmic staining. **c** CD105 stain. More even cytoplasmic staining

We observed that both markers stained endothelial cells quite specifically. vWF had a strong and granular cytoplasmic staining pattern, whereas CD105 had a more even and occasionally weaker cytoplasmic stain (Fig. 2). Generally, little background staining was observed, except in a few vWF sections. The distributions of both vWF-MVD and CD105-MVD were skewed, and the median vWF-MVD was 15.5 per field (range 0.7–62.0) and the median CD105-MVD was 12.7 (range 0.7–50.0).

### Relationships between MVDs of the endothelial markers

**Fig. 3:**
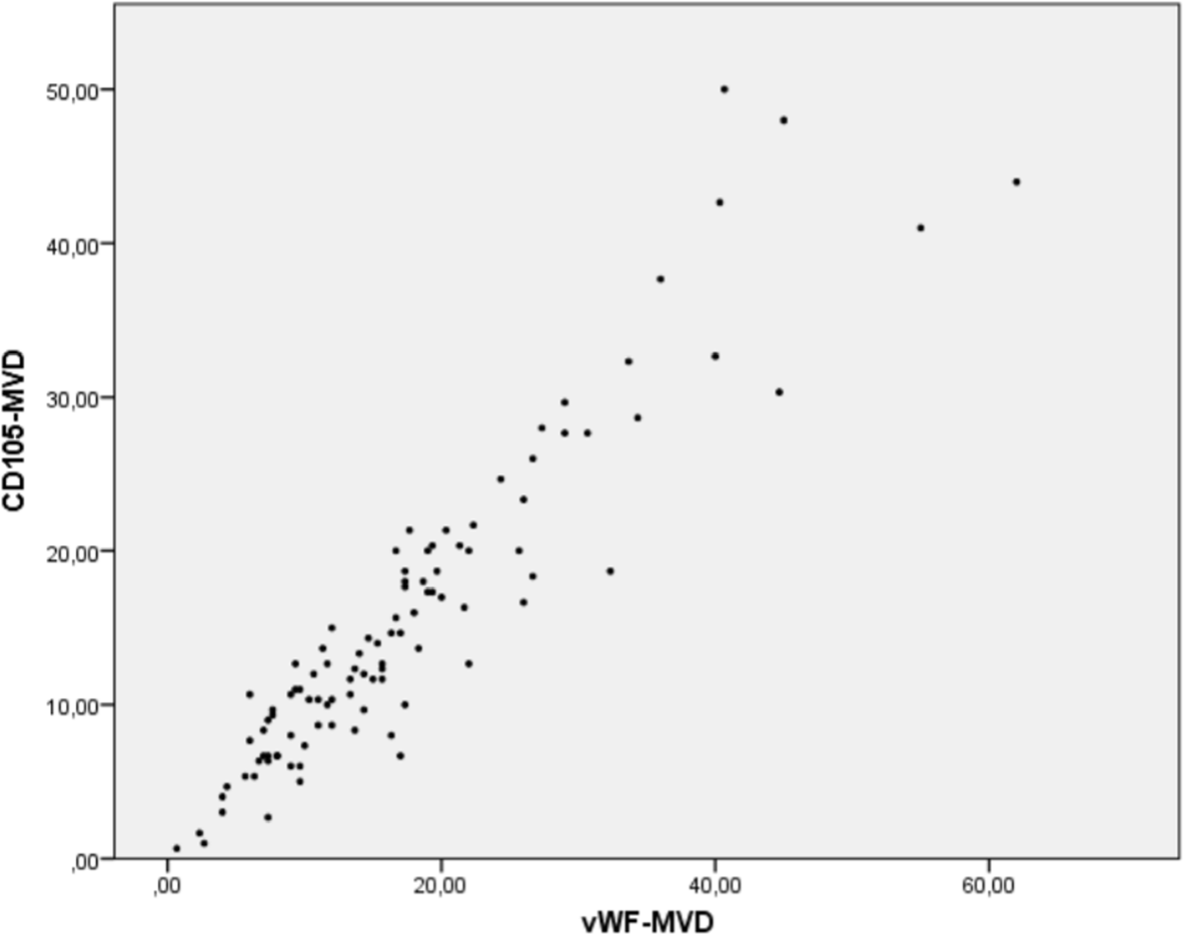
Scatterplot of vWF-MVD and CD105-MVD. The scatterplot shows the close correlation between the markers, with dots forming a close-to linear pattern in line with the high correlation coefficient (ρ = 0.92). However, the spread increases for higher MVD counts

vWF-MVDs and CD105-MVDs were significantly correlated (*p* < 0.001, ρ = 0.92). A scatterplot showing the relationship is found in Fig. 3. The median CD105-MVD/vWF-MVD ratio was 0.91 with 95% confidence interval (0.87, 0.95).

### MVD and histopathology

Of the features previously found to significantly associate with faster growth in univariable analyses (thromboses, high cellular density, and mitotic count) [13], only mitotic count was significantly associated with any MVD in this study (Table 1). However, CD105-MVD tended to be higher in cases with present thromboses or high cellular density despite the non-significant associations. The same tendency was observed for vWF-MVD and thromboses (Table 1). Interestingly, both MVDs were significantly associated with subjectively evaluated high vascular densities on HE sections.

**Table 1:** Associations between MVDs and histopathological features

Histopathological features^a^	vWF-MVD (Median, 95% CI, ρ)	CD105-MVD (Median, 95% CI, ρ)
Thromboses	*p* = 0.160	*p* = 0.125
- Not present	12.0 (8.0, 21.8)	9.3 (7.2, 17.2)
- Present	15.7 (15.5, 20.2)	13.3 (13.9, 18.4)
High cellular density	*p* = 0.345	*p* = 0.082
- Not present	14.7 (14.0, 19.3)	12.0 (12.1, 16.8)
- Present	17.3 (14.6, 23.2)	16.3 (13.9, 21.9)
Mitotic count^b^	*p* = 0.004*	*p* = 0.001*
	ρ = 0.29	ρ = 0.33
High vascular density	*p* = 0.016*	*p* = 0.004*
- Not present	13.5 (12.9, 18.5)	11.3 (11.3, 15.8)
- Present	17.5 (16.5, 24.0)	16.5 (15.2, 22.9)

*vWF-MVD* Microvessel density count of von Willebrand factor. *CD105-MVD* Microvessel density count of CD105. *CI* Confidence interval. *p*: *p*-value. *ρ* Spearman correlation coefficient. ^a^ Subjectively assessed on hematoxylin-eosin sections. ^b^ Counted in hotspots for 10 high-power fields. *Statistically significant associations, *p* < 0.05

### MVD and growth

**Fig. 4:**
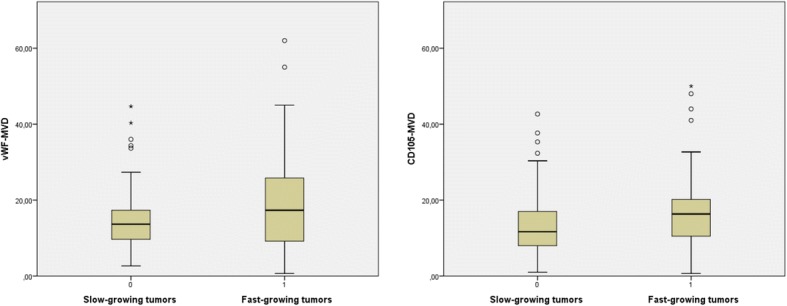
Boxplots of vWF-MVD and CD105-MVD in slow and fast-growing tumors. Both MVDs show tendencies towards higher counts in fast-growing tumors, however, the spreads are large within both growth groups

In this study, only CD105-MVD was significantly associated with faster tumor growth in the univariable analyses (Table 2). However, the ranges of both MVDs were quite wide within both fast and slow-growing tumors (Table 2, Fig. 4). Nevertheless, CD105-MVD was no longer significant in a multivariable model including thromboses and high cellular density, where the two latter features were significant independent predictors of faster growth (Table 3).

**Table 2:** Univariable analyses of associations between MVDs and tumor growth, Mann-Whitney U tests

	Slow-growing tumors	Fast-growing tumors	*p*-values
vWF-MVD	Median: 13.7 95% CI (12.9, 18.2)	Median: 17.3 95% CI (15.5, 22.7)	*p* = 0.211
	Range: 2.7–44.7	Range: 0.7–62.0	
	*N* = 49	*N* = 52	
CD105-MVD	Median: 11.7 95% CI (11.0, 16.2)	Median: 16.3 95% CI (14.2, 20.5)	*p* = 0.049*
	Range: 1.0–42.7	Range: 0.7–50.0	
	*N* = 50^a^	*N* = 51^a^	

*vWF-MVD* Microvessel density count of von Willebrand factor. *CD105-MVD* Microvessel density count of CD105. *CI* Confidence interval. *N* Number of cases. * Statistically significant associations, *p* < 0.05. ^a^ One case was excluded from the vWF-MVD assessment, and another from the CD105-MVD assessment. These two cases were in different growth groups, which caused the change in number of cases for each group

**Table 3:** Multivariable binary logistic regression analysis of morphologic features and faster growth

Morphological features	Multivariable odds ratio (95% CI)	Multivariable *p*-values
High cellular density^a^	2.55 (1.007, 6.475)	0.048*
Thromboses^a^	4.21 (1.245, 14.253)	0.021*
CD105-MVD	1.03 (0.982, 1.070)	0.255

*CI* Confidence interval. *CD105-MVD* Microvessel density count of CD105. ^a^Subjectively assessed on hematoxylin-eosin sections. *Statistically significant associations, *p* < 0.05

## Discussion

In this study, CD105-MVD, and not vWF-MVD, was significantly associated with faster growth in the univariable analyses. However, the relation was lost when adjusted for the presence of thromboses and high cellular density in a multivariable model, where these two latter features were significant independent predictors of faster growth. Both MVDs associated significantly with mitotic count, but neither with the presence of thromboses nor high cellular density.

Biological reasons for why some GBMs grow faster than others are sparsely studied in human patients, which is mainly due to difficulties in acquiring in vivo pretreatment growth estimates [10, 11]. In addition, most research on growth processes have been conducted on in vitro or animal models which fail to accurately imitate the unique micro-milieu of the human GBM [30]. Moreover, by having preoperative growth as an outcome variable instead of overall survival, we avoid the effects of clinical factors found to be influential on survival, such as age at diagnosis, tumor size at diagnosis, Karnofsky performance status, comorbidity, and effects of treatment. Corticosteroid treatment was the only preoperative treatment received by our patients (82 patients); however, it was not significantly associated with tumor growth when corrected for tumor volume [10], and associations between histopathology and growth were independent of such treatment [13]. Altogether, these aspects of the study made it possible to study the biology of GBM growth as unaffected as clinically justifiable.

### Endothelial markers and angiogenesis

The prognostic role of MVD measurements in glioblastomas is unclarified [31–35]. However, a few studies have reported that vWF-MVD and CD105-MVD may predict the malignancy grade and prognosis of gliomas [36, 37]. The finding that only CD105-MVD was significantly associated with growth in the univariable analyses, is in line with other univariable studies which have found more promising results for CD105-MVD than for CD31-MVD (a pan-endothelial marker) as prognostic markers of GBM [33, 34]. These studies speculated the potential prognostic inferiority of pan-endothelial markers (i.e vWF, CD31, CD34) was caused by the additional staining of pre-existing angiogenically inactive vessels [23]. In contrast, many studies have shown that CD105 predominantly stain proliferating endothelial cells [22, 33, 38–46]. However, a few studies have observed CD105-positive vessels in normal [47] and GBM-adjacent brain tissue [48], and the marker needs further validation. Moreover, it has been shown that vWF sometimes fail to stain microvessels in both normal and neoplastic tissue [46].

vWF-MVD and CD105-MVD were highly correlated (Fig. 3), with a higher median and upper range for vWF-MVD. In addition, the high CD105-MVD/vWF-MVD ratio suggests most vasculature of GBM are angiogenically active. Because the markers were counted in corresponding HPFs, a high correlation coefficient was expected. However, some of the differences in the MVDs could be caused by random variations in vascular densities of different sections and the granular staining of vWF (Fig. 2), which sometimes made it more difficult to distinguish separate vascular units than in CD105 sections.

### Neovasculature and tumor growth

CD105-MVD was no longer significantly associated with preoperative tumor growth in a multivariable model with thromboses and high cellular density, where both latter features were significant independent predictors of faster growth (Table 3). However, reverse causation may also be possible: faster growth could lead to thromboses and high cellular density. Furthermore, we observed that fast-growing tumors could have quite low CD105-MVD scores and slow-growing quite high (Table 2, Fig. 4), which was in line with the finding that CD105-MVD explained very little of the variance in speed of growth (3%) in the univariable analysis (data not shown). Similar ranges of CD105-MVD were observed within the growth groups when patients with sparse tissue amounts (46 cases) were excluded (Additional file 1), and the weak association was thus unlikely a result of sampling errors. Altogether, our results suggest that vWF-MVD and CD105-MVD are not predictive of faster GBM growth.

There are several biological mechanisms which could potentially explain the inferiority of CD105-MVD as an independent predictor of tumor growth. One reason could be that tumors can create a surplus of or ineffective vasculature due to excessive angiogenic stimuli [23], potentially leading to an overrepresentation of MVD counts. Excessive angiogenic stimuli may be caused by oncogenic mutations (known as hypoxia-independent angiogenesis [49]). Other explanations could be that other mechanisms of glioma-associated neovascularization account for additionally needed vasculature in fast-growing tumors [49], such as vascular co-option [50], vasculogenesis [51, 52], vascular mimicry (non-endothelial vasculature) [53], and glioblastoma-endothelial cell transdifferentiation [42]. Vascular mimicry is the process most likely to be overlooked by our methodology due to the lack of endothelial cells. In addition, the presence of vascular mimicry has been found to significantly predict higher glioma grades and worse prognosis [53]. However, it is uncertain to which degree and how the different processes of neovascularization interact, and further studies are needed [49].

In our previous study, we speculated that hypoxia initiated by thromboses facilitated growth through an induction of angiogenesis [13]. However, the finding that the presence of thromboses was still a significant independent predictor of faster tumor growth when the degree of angiogenesis was not, suggests angiogenesis-independent mechanisms driven by hypoxia contribute to faster GBM growth. Such hypoxia induced mechanisms may act through other mechanisms of glioma-associated vascularization [49], augmentation of proliferation [54, 55], and initiation of invasiveness [19, 55]. Increased invasiveness is one of the proposed mechanisms of resistance to anti-VEGF (bevacizumab) treatment [55–59], and thus the lack of survival benefit in randomized trials [20, 21]. Some studies even suggest GBM growth is possible without an induction of angiogenesis [19, 60]. Such angiogenesis-independent growth may be described by the “go-or-grow” hypothesis, where tumor cells switch between two mutually exclusive phenotypes of either proliferative or invasive characteristics [61, 62]. Hypoxia has been proposed to induce the switch to the invasive phenotype [62]. In this way, hypoxic tumor cells migrate away from hypoxic areas and switch back to a proliferative phenotype when nutrients are adequate without an induction of angiogenesis [60]. In addition, Sakariassen et al. [19], discovered that xenotransplanted GBMs in nude rats could present as fatal diseases without signs of angiogenesis. Nevertheless, invading cells are unlikely to be captured as contrast enhancement without an induction of angiogenesis [19, 59, 63], and are therefore unlikely to have been measured in our study. Additionally, the non-significant multivariable association for CD105-MVD could perhaps be caused by the nearly significant associations between CD105-MVD and thromboses and high cellularity (Table 1). Collectively, our findings support that angiogenesis-independent mechanisms driven by hypoxia contribute to faster GBM growth, which might explain the lack of survival benefit of anti-VEGF treatment.

As thromboses, high cellular density maintained its role as a significant independent predictor of faster growth in our study (Table 3). This finding substantiates our previous speculation that it is a better marker of high proliferation rates than high mitotic counts, because mitotic count has many potential sources of errors [64], and higher counts were significantly associated with the presence of thromboses [13] and increasing CD105-MVD counts (Table 1).

### Microvessel methodology

So far, there is no standard method for quantification of MVD, however, initiatives on international standardizations have been made [65]. Like in our study, most studies are based on the methods described by Weidner et al. [27] with modifications: they count single positive cells and avoid areas of sclerosis, necrosis, and non-neoplastic tissue. However, few have specified their handling of glumeruloid tufts and longer vessels. We believe as Leon et al. [36], that counting a glomeruloid tuft as one vascular unit might underestimate the angiogenic stimuli of the tumor. Furthermore, the subjective assessment of hotspots and interpretation of positive immunostaining give rise to problematic inter-observer variability, which has been reported as quite high for MVD assessments in GBMs [66].

Even though we found significant associations with both MVDs and subjectively assessed high vascular densities on HE slides, the spreads of the MVDs were wide within and overlapping between the categories of vascular density (Table 1). These findings were in line with the fact that capillaries is known to be inconspicuous on HE slides [28].

### Strengths and limitations

Limitations of the assessments of tumor volumes on MRI scans, growth rates, and histopathological features have previously been described in detail [10, 13]. The main strengths are the relatively large number of patients with a population based referral and the reproducibility assessment of tumor volumes [10]. Potential biases are selection biases, preoperative steroid treatment, differences in diagnostic MRI scanners, different timing and administration of the contrast agent, tumor cells existing beyond the contrast enhancing rim [67, 68], and sampling errors and inter-observer variability of the histopathological assessments. Additionally, our analyses were exploratory and should be validated in future studies.

## Conclusions

Our results showed that MVD assessments of vWF and CD105 were not independent predictors of radiological speed of growth, although CD105-MVD was significantly associated with faster growth in the univariable analysis. In contrast, thromboses and high cellular density were significant independent predictors of faster growth. In summary, our findings suggest angiogenesis-independent mechanisms contribute to faster GBM growth.

## Additional files

Additional file 1: Univariable analysis of associations between CD105-MVD and tumor growth when cases with sparse tissue amount are excluded (46 cases). Mann-Whitney U tests. CD105-MVD: Microvessel density count of CD105. CI: Confidence interval. N: Number of cases. (DOCX 12 kb)

Additional file 2: Dataset supporting conclusions. The minimal dataset necessary to replicate the findings reported in the article. Microvessel densities (MVDs) of both markers are presented as mean counts for 3 high-power fields (HPFs). Regarding the categorical histopathological features (thromboses, high cellular density, and high vascular density), “1” indicates that features are present, while “0” indicates that they are not. Mitotic counts are counted in 10 HPFs. Tumors growing faster than expected are indicated by “1”, whereas slow-growing tumors are indicated by “0”. Patients preoperatively treated with corticosteroids or having sparse amount of tissue available for the histopathological evaluation are indicated by “1” in the respective variables. (XLSX 15 kb)

## Abbreviations

CD: Clusters of differentiation
FFPE: Formalin-fixed paraffin-embedded
GBM: Glioblastoma
GFAP: Glial fibrillary acidic protein
HE: Hematoxylin-eosin
HPF: High-power field
HRP: Horseradish peroxidase
IDH1: Isocitrate dehydrogenase 1
LSAB: Labeled streptavidin-biotin
MRI: Magnetic resonance imaging
MVD: Microvessel density
OR: Odds ratio
VEGF: Vascular endothelial growth factor
vWF: von Willebrand factor
WHO: World Health Organization
