# Supplementary material for: Angiogenesis and radiological tumor growth in patients with glioblastoma
Source: BMC Cancer. 2018 Sep 3;18:862. doi: 10.1186/s12885-018-4768-9 (PMC6122710; doi:10.1186/s12885-018-4768-9)
Supplement: Supplementary file 1 — Univariable analysis of associations between CD105-MVD and tumor growth when cases with sparse tissue amount are excluded (46 cases). Mann-Whitney U tests. CD105-MVD: Microvessel density count of CD105. CI: Confidence interval. N: Number of cases. (DOCX 12 kb) [file 12885_2018_4768_MOESM1_ESM.docx]

**Additional file 1**

|  | Slow-growing tumors | Fast-growing tumors |
| --- | --- | --- |
| CD105-MVD | Median: 12.3 95% CI (12.2, 19.2)  Range: 6.0 – 42.7  N = 31 | Median: 17.7 95% CI (15.5, 24.4)  Range: 1.7 – 50.0  N = 25 |
